# Supplementary material for: Loneliness among people with severe mental illness during the COVID-19 pandemic: Results from a linked UK population cohort study
Source: PLoS One. 2022 Jan 13;17(1):e0262363. doi: 10.1371/journal.pone.0262363 (PMC8757957; doi:10.1371/journal.pone.0262363)
Supplement: Attachment — Submitted filename: review report ploseone.docx [file pone.0262363.s001.docx]

**Review report**

The topic of research is very interesting and it needs to be published. Nevertheless, I would like to raise the following issues which I considered is good to be taken for the betterment of the paper.

**GENERAL**

**Title:** I personally recommend you to take minor revision on your title of the research because it is more comfortable for readers if it can be revised as ‘Loneliness among people with severe mental illness during the COVID-19 pandemic: results from a linked UK population cohort study”

**Abstract**

**Purpose:** The title of your research needs to have complete information about what you are going to do. Your title is not sufficient to describe what is mentioned in your purpose of study section. It needs revision; put your exact objective with respect to your topic of interest.

**Introduction:** interesting

**Methods:** interesting

**Result:** Table 2 is out of what you are intended to do; beyond your objective

**Discussion:** very interesting but too little when compared to what you have reported in the result section and inadequate scientific evidences. Needs revision

**References:** there are a number of web link citations in the main document; it is good if you use a uniform referencing style.
