# Supplementary material for: Loneliness among people with severe mental illness during the COVID-19 pandemic: Results from a linked UK population cohort study
Source: PLoS One. 2022 Jan 13;17(1):e0262363. doi: 10.1371/journal.pone.0262363 (PMC8757957; doi:10.1371/journal.pone.0262363)
Supplement: Attachment — Submitted filename: Response to Reviewers.docx [file pone.0262363.s002.docx]

Dear Plos Editorial team and reviewers,

Thank you for processing the submitted manuscript and for providing us with the opportunity to revise the submitted manuscript. We thank the reviewers for their thoughtful comments which have helped us to revise and improve the manuscript.

We have addressed each of the reviewers’ comments and expanded the manuscript’s discussion section. Please find our detailed responses to each of the comments below in blue text. We hope that the manuscript may now be suitable for publication, but please do not hesitate to contact us if there are matters to address, or if we can provide additional information.

Kind wishes,

Paul Heron

**Comments to the Author**

Considering the reviewers comments and my own reading of the paper, I am suggesting a major revision for this paper. Third reviewer who has not submitted full review but saved partial review with following comment.
"The study is timely but lacks methodological and statistical rigor", try to respond to this comment as well.
Author response: We have addressed a problem with the study data being publicly available and a problem with the analysis plan in the comments below. The discussion section has been expanded and adds context to the timing of data collection. These points are described in further detail below in response to reviewer comments.

1. Is the manuscript technically sound, and do the data support the conclusions?

The manuscript must describe a technically sound piece of scientific research with data that supports the conclusions. Experiments must have been conducted rigorously, with appropriate controls, replication, and sample sizes. The conclusions must be drawn appropriately based on the data presented.

Reviewer #1: Yes

Reviewer #2: Yes

Author response: Thank you

2. Has the statistical analysis been performed appropriately and rigorously?

Reviewer #1: Yes

Reviewer #2: Yes

Author response: Thank you

3. Have the authors made all data underlying the findings in their manuscript fully available?

The [PLOS Data policy](http://www.plosone.org/static/policies.action#sharing) requires authors to make all data underlying the findings described in their manuscript fully available without restriction, with rare exception (please refer to the Data Availability Statement in the manuscript PDF file). The data should be provided as part of the manuscript or its supporting information, or deposited to a public repository. For example, in addition to summary statistics, the data points behind means, medians and variance measures should be available. If there are restrictions on publicly sharing data—e.g. participant privacy or use of data from a third party—those must be specified.

Reviewer #1: Yes

Reviewer #2: No

Author response: We are not able to share a de-identified data set as we do not have consent from the research participants to do this. We have checked with the GDPR team at the University of York and they have advised us that we cannot upload this data to a public repository without explicit consent from the study participants. Data requests for the full dataset may be sent to the Closing the Gap Network email: [ctg-network@york.ac.uk](mailto:ctg-network@york.ac.uk) whose steering committee manage our data requests.

4. Is the manuscript presented in an intelligible fashion and written in standard English?

PLOS ONE does not copyedit accepted manuscripts, so the language in submitted articles must be clear, correct, and unambiguous. Any typographical or grammatical errors should be corrected at revision, so please note any specific errors here.

Reviewer #1: Yes

Reviewer #2: Yes

Author response: Thank you

5. Review Comments to the Author

Please use the space provided to explain your answers to the questions above. You may also include additional comments for the author, including concerns about dual publication, research ethics, or publication ethics. (Please upload your review as an attachment if it exceeds 20,000 characters)

Reviewer #1: The topic of research is very interesting and it needs to be published. Nevertheless, I would like to raise the following issues which I considered is good to be taken for the betterment of the paper.
GENERAL
Title: I personally recommend you to take minor revision on your title of the research because it is more comfortable for readers if it can be revised as ‘Loneliness among people with severe mental illness during the COVID-19 pandemic: results from a linked UK population cohort study”

Author response: We have updated the title as recommended
Abstract
Purpose: The title of your research needs to have complete information about what you are going to do. Your title is not sufficient to describe what is mentioned in your purpose of study section. It needs revision; put your exact objective with respect to your topic of interest.

Author response: We have updated the ‘Purpose’ section of the abstract to provide a more complete picture of the manuscript. Taken with the above comment that suggests a minor amendment to the title, we believe that this comment refers to the ‘Purpose’ section of the abstract rather than the manuscript title. We apologise if we have misinterpreted this comment and would be happy to amend the manuscript title further.
Introduction: interesting
Methods: interesting
Result: Table 2 is out of what you are intended to do; beyond your objective

Author response: Thank you for highlighting this. The analysis plan contains an error where it refers to the variable as ‘Social isolation’ rather than ‘Perceived social support’. This can be corroborated by the survey being uploaded prior to analysis which contains a measure of Perceived social support, but no measure of Social isolation (link to survey in analysis plan: https://osf.io/8qwhd/#!). We have added information to the ‘Analysis’ section of the manuscript to clarify this.
Discussion: very interesting but too little when compared to what you have reported in the result section and inadequate scientific evidences. Needs revision

Author response: We have expanded the discussion section. In particular, we discuss two points, mentioned in the below comments; the internet and digital connectivity for social contact, and adding context to when the data was collected in relation to pandemic restrictions.
References: there are a number of web link citations in the main document; it is good if you use a uniform referencing style.

Author response: We have updated the in-text weblinks to maintain consistency

Reviewer #2: I am very grateful to the Editors that I have the opportunity to revise this manuscript.

Below is my review report:

-The statistic analyzes are well done.

Formal problem:

-IMDD: the meaning of the abbreviation is not explained in the text. If it is Index of Multiple Deprivation, than in Table 1 there is „moderate deprivation” and inTable 2,3 the same category called „medium”. It will be better to check nomenclature and use one name.

Author response: We have updated the text to explain the abbreviation, and to refer to it as Index of Multiple Deprivation (IMD), rather than Index of Multiple Deprivation Decile (IMDD) which was incorrect. The text also now consistently refers to the middle category as ‘moderate’ deprivation.

-There is a reference form error on page 10: Mishu 2019- I can not find in reference list, and the form is problematic in the text.

Author response: We have updated the reference list and article body to add the Mishu 2019 reference. Other references have also been re-numbered to allow this.

Questions or suggestions about method:

-When did you do the reports? I think it can be important, that study sampling was in first “COVID pandemic wave” (2020 spring) or after half/ one year from its beginning. I think mental status could be deterioration after several months of lockdown.
It is interesting finding, regarding the timing of research: “Only a minority of participants reported a worsening to their financial wellbeing or reduction in professional activity during the pandemic.This may be because people with SMI were already disproportionately affected by socioeconomic deprivation and unemployment prior to the pandemic.” Now, among the waves of the COVID pandemic of the past 1,5 years, is this research result still the same or only at the beginning of the pandemic was?
Author response: Thank you for this. We have added detail to the discussion to add context to these findings and discuss how long-term effects of restrictions may have influenced the findings.

-In this study severe mental ill health is treated as a homogeneous group, it is a question for me that patients with different type of mental illnesses (and different coping ability or pss) can be treat like a homogeneous group.

Author response: We were also interested to explore differences between diagnoses but did not want to conduct too many post-hoc analyses. We have now added detail to the ‘Limitations’ section to discuss this.

-I would have also asked in questionare about (online) varieties of social support because just as the general population has replaced personal relationships with this, I think study group as well.

Author response: Thank you, we have now added detail to the discussion about the role of internet and digital devices in social communication during the pandemic among people with SMI. Digital connectivity and internet use was explored as part of the OWLS study in a separate article, so we have referred readers to these articles for further information.


Discussion:
As I read the results and discussion, I felt that the study has the result what I expected at beginning of the introduction part. With the research results, the authors fulfilled the described study goal regarding the explore perceived social support and loneliness and factor associations during the Covid-19 pandemic, but there was no surprising finding at the end. I would have liked to read about what coping methods were, and in more detail what deterioration of loneliness was associated with their patients mental health in lockdown period. Maybe the study questionaire was not enough detailed or targetted, and that is why there is not a breakthrough result.

Author response: Thank you. Due to the continually changing nature of the pandemic, and how concerning we found these findings to be, we felt it was important to publish these results early. We hope they will generate interest and discussion around the topic. In time, we plan to publish the OWLS study’s follow-up data to explore long-term trends in loneliness and social support, and to explore these topics with qualitative methods.
Conclusion:
“Additional study is also needed to understand the barriers to social connectivity for people with SMI, and to understand the best strategies to intervene.” Correct, and important conclusion. I hope you will continue your study and we will read about the barriers to social connectivity and your strategies to intervene.

Author response: Thank you.

6. PLOS authors have the option to publish the peer review history of their article ([what does this mean?](https://journals.plos.org/plosone/s/editorial-and-peer-review-process#loc-peer-review-history)). If published, this will include your full peer review and any attached files.


If you choose “no”, your identity will remain anonymous but your review may still be made public.


**Do you want your identity to be public for this peer review?** For information about this choice, including consent withdrawal, please see our [Privacy Policy](https://www.plos.org/privacy-policy).

Reviewer #1: **Yes:** MENGESHA SRAHBZU BIRESAW

Reviewer #2: No
