# Supplementary material for: Loneliness among people with severe mental illness during the COVID-19 pandemic: Results from a linked UK population cohort study
Source: PLoS One. 2022 Jan 13;17(1):e0262363. doi: 10.1371/journal.pone.0262363 (PMC8757957; doi:10.1371/journal.pone.0262363)
Supplement: Attachment — Submitted filename: Response to reviewers 20_12_21.docx [file pone.0262363.s003.docx]

PONE-D-21-22036R1

Loneliness among people with severe mental illness during the COVID-19 pandemic: results from a linked UK population cohort study

PLOS ONE

Dear PLOS ONE Editorial team and Reviewers,

On behalf of the authors, thank you to the editors and reviewers. We appreciate your feedback to refine and improve the article. We have updated the article in line with the recommendations. Please see our responses below in blue text.

We hope that the article may be ready for acceptance. Please do not hesitate to contact me if you would like to discuss the manuscript further.

Kind wishes,
Paul

**Feedback from PLOS ONE**

Journal Requirements:

Please review your reference list to ensure that it is complete and correct. If you have cited papers that have been retracted, please include the rationale for doing so in the manuscript text, or remove these references and replace them with relevant current references. Any changes to the reference list should be mentioned in the rebuttal letter that accompanies your revised manuscript. If you need to cite a retracted article, indicate the article’s retracted status in the References list and also include a citation and full reference for the retraction notice.

Author response: The reference list has been updated to correct errors.

Additional Editor Comments:

Considering the reviewers suggestion, I am going with a decision of minor revision.


[Note: HTML markup is below. Please do not edit.]

Reviewers' comments:

Reviewer's Responses to Questions

**Comments to the Author**

1. If the authors have adequately addressed your comments raised in a previous round of review and you feel that this manuscript is now acceptable for publication, you may indicate that here to bypass the “Comments to the Author” section, enter your conflict of interest statement in the “Confidential to Editor” section, and submit your "Accept" recommendation.

Reviewer #1: All comments have been addressed

Reviewer #2: All comments have been addressed

2. Is the manuscript technically sound, and do the data support the conclusions?

The manuscript must describe a technically sound piece of scientific research with data that supports the conclusions. Experiments must have been conducted rigorously, with appropriate controls, replication, and sample sizes. The conclusions must be drawn appropriately based on the data presented.

Reviewer #1: Yes

Reviewer #2: Yes

3. Has the statistical analysis been performed appropriately and rigorously?

Reviewer #1: Yes

Reviewer #2: Yes

4. Have the authors made all data underlying the findings in their manuscript fully available?

The [PLOS Data policy](http://www.plosone.org/static/policies.action#sharing) requires authors to make all data underlying the findings described in their manuscript fully available without restriction, with rare exception (please refer to the Data Availability Statement in the manuscript PDF file). The data should be provided as part of the manuscript or its supporting information, or deposited to a public repository. For example, in addition to summary statistics, the data points behind means, medians and variance measures should be available. If there are restrictions on publicly sharing data—e.g. participant privacy or use of data from a third party—those must be specified.

Reviewer #1: Yes

Reviewer #2: Yes

5. Is the manuscript presented in an intelligible fashion and written in standard English?

PLOS ONE does not copyedit accepted manuscripts, so the language in submitted articles must be clear, correct, and unambiguous. Any typographical or grammatical errors should be corrected at revision, so please note any specific errors here.

Reviewer #1: Yes

Reviewer #2: Yes

6. Review Comments to the Author

Please use the space provided to explain your answers to the questions above. You may also include additional comments for the author, including concerns about dual publication, research ethics, or publication ethics. (Please upload your review as an attachment if it exceeds 20,000 characters)

Reviewer #1: THANK YOU very much for addressing my concerns for the betterment of the paper. in its current status it has became an interesting paper.

Reviewer #2: Dear Author!

Thank you for your revised manuscript and answers for my questions.

Your manuscript is still timely, but need minor revision.

My review report:

Technical problem:
Reference 33: There are 9 authors mentioned, and as I know you should write max. 6 then abbreviation et al.- Please look after it, and correct.

Author response: This has been corrected.

1. Abstract: Purpose: Aim/ goal/ purpose - the term- should be use in drafting this part of manuscript - please revise (content is ok)

Author response: The terms in this section have been updated.

2. Introduction, result part do not need further revision- in my opinion.

Author response: Thank you.

3. Methods: Data collection timing should be here mentioned firstly.

Author response: The methods have now been updated with the recruitment and data collection timing.

4. Discussion
Discussion needs revision. The main message of your study and the results should be in focus, and should be detailed here. Now the new paragraphs seem to be too detailed- as I see the whole discussion, and the results. Conditions of data collection and its timing need to be shorten here. Use of internet by SMI patient is very interesting, and it is good to read about it, but not involved in this study- so it is an additional information, need compress too.

Author response: The discussion section has had some detail removed to make it more succinct. This will also mean that the focus of the discussion is placed more on the main findings.
